# G6PC indicated poor prognosis in cervical cancer and promoted cervical carcinogenesis in vitro and in vivo

**DOI:** 10.1186/s12958-022-00921-6

**Published:** 2022-03-11

**Authors:** Kun Zhu, Chunling Deng, Pan Du, Taorui Liu, Junjie Piao, Yingshi Piao, Meng Yang, Liyan Chen

**Affiliations:** 1 Cancer Research Center, Yanbian University Medical College, Gong Yuan Road No.977, Yanji, 133002 China; 2 Key Laboratory of the Science and Technology Department of Jilin Province, Yanji, China; 3 Department of Physiology, Medicine College, Jingchu University of Technology, Jingmen, 448000 China

**Keywords:** G6PC, Cervical cancer, Prognosis, EMT, Angiogenesis, PI3K/AKT pathway

## Abstract

**Background:**

The glucose-6-phosphatase catalytic subunit (G6PC) is a key enzyme that is involved in gluconeogenesis and glycogen decomposition during glycometabolism. Studies have shown that G6PC is abnormally expressed in various cancers and participates in the proliferation and metastasis of tumors. However, the role of G6PC in cervical cancer remains poorly established.

**Methods:**

To analyze the expression of G6PC in cervical cancer tissues in patients by immunohistochemistry. Effects of G6PC deregulation on cervical cancer phenotype were determined using MTT, colony formation, transwell, and wound-healing assays. And constructed a nude mouse xenograft tumor model and CAM assay in vivo. The effect of G6PC on glycolysis in cervical cancer was also evaluated. Effect of G6PC on PI3K/AKT/mTOR pathway was detected by Western blot assay.

**Results:**

In this study, G6PC expression was found to be upregulated in cervical cancer tissues, and this upregulated expression was associated with LN metastasis, clinical stage, recurrence, and disease-free survival and overall survival rates, indicating that G6PC could serve as a novel marker of early diagnosis in cervical cancer. G6PC promoted proliferation, invasion, epithelial mesenchymal transition (EMT) progression, and angiogenesis of cervical cancer cells. Mechanistically, G6PC activated PI3K/AKT/mTOR pathways. The PI3K/AKT pathway inhibitor, LY294002 could partially attenuate the effect.

**Conclusions:**

G6PC plays a key role in the progression of cervical cancer, and overexpressed G6PC is closely related to patient LN metastasis, clinical stage, recurrence and shortened survival. G6PC promoted cervical cancer proliferation, invasion, migration, EMT progression, and angiogenesis, partially through activating the PI3K/AKT pathway. G6PC, as a metabolic gene, not only plays a role in metabolism, but also participates in the development of cervical cancer. Its complex metabolic and non metabolic effects may be a potential therapeutic target and worthy of further study.

## Introduction

Cervical cancer (CC) is the most common malignant tumor in women worldwide. The most recent data show that cervical cancer is still the second leading cause of cancer-related death among women aged 20 to 39 years [1]. The incidence of CC is predominantly in developing countries and the overall of 5-year survival rate is relatively low [2]. Human papillomavirus (HPV) infection is a major risk factor for cervical cancer [3]. At present, the main methods for treating CC are surgery, radiotherapy and chemotherapy, but the overall prognosis of patients with metastatic or recurrent disease is still poor [4, 5]. Therefore, there is an urgent elucidate further clarify the potential biological mechanisms of CC in order to obtain better treatment strategies.

The catalytic subunit of glucose 6 phosphatase (G6PC), which is located on the endoplasmic reticulum membrane, is one of the key enzymes in the regulation of glucose homeostasis and glycogenolysis, and G6PC catalyzes the final step of the gluconeogenesis and glycogenolysis pathway [6]. Studies have shown that G6PC expression is low in gluconeogenic tissue tumors such as hepatocellular carcinoma and clear cell renal cell carcinoma [7, 8], but G6PC is overexpressed in nongluconeogenic tissue tumors such as ovarian cancer and glioblastoma [9, 10]. In ovarian cancer or glioma, G6PC exerts carcinogenic effect, which regulates the cell cycle and EMT progress of ovarian cancer cells. In glioblastoma, G6PC can counteract the inhibition of glycolysis by 2DG and restore the aggressiveness of tumor cells [9, 10]. G6PC exerts its complex metabolic and nonmetabolic effects as a result of its abnormal expression in many types of tumors, making G6PC a potential therapeutic target. To date, the role of G6PC in CC remains unclear.

In this study, we investigated the expression of G6PC in CC cells, and explored the relationship between G6PC expression and clinicopathological characteristics. In addition, we analyzed the effects of G6PC on cell growth, metastasis and angiogenesis. These findings indicate that G6PC can accelerate the proliferation, and metastasis of CC by activating the PI3K/AKT/mTOR signaling pathway.

## Materials and methods

### Clinical samples

We randomly selected 93 cervical cancer and 24 adjacent normal cervical tissue samples from Shanghai Outdo Biotech Co., Ltd. The samples included patients who underwent surgery between January 2010 and October 2011, with rigorous follow-up for survival status. The inclusion and selection criteria of patients, including no other treatment intervention, were received before admission, and all patients met the indications for surgical resection. Clinical information on the samples is summarized in Tables 1 and 2. The ages of patients ranged from 29 to 70. In accordance with the 7th edition of the American Joint Committee on Cancer, 74 of 93 CC specimens were determined as early stage (I-II) and 19 as late stage (III-IV). 18 specimens had LN metastasis, 75 were negative. All patients were followed up for more than 6 years after surgery.

**Table 1:** Expression of G6PC protein in cervical cancer

Diagnosis	No.of case	G6PC				Positive case rate (%)	Strong positive case rate (%)
		**-**	** + **	** + + **	** + + + **		
Cervical cancer	93	15	17	30	31	83.9**	65.6**
Adjacent normal	24	16	6	2	0	33.3	8.3

**Positive rate**: percentage of positive cases with + , +  + , and +  +  + staining score

**Strongly positive rate** (high-level expression): percentage of positive cases with +  + and +  +  + staining score

^**^ *p* < 0.01 compared with adjacent normal cervical

**Table 2:** Relationship between G6PC overexpression and the clinicopathological features of cervical cancer

Variables	No. of case (*n*)	G6PC strong positive rate (%)	χ^2^	*P* value
**Age (years)**			0.420	0.517
< 46	45	31 (68.9)		
≥ 46	48	30 (62.5)		
**Tumor invasion depth**			3.668	0.055
T1-T2	74	45 (60.8)		
T3-T4	19	16 (84.2)		
**LN metastasis**			5.368	0.021*
Positive	18	16 (88.9)		
Negative	75	45 (60)		
**Histological grade**			0.998	0.318
Grade 1–2	26	15 (57.7)		
Grade-3	67	46 (68.7)		
**Clinical stage**			9.963	0.007**
I	46	23 (50)		
II	28	22 (78.6)		
III +IV	19	16 (84.2)		
**Recurrence**			10.069	0.002**
Yes	35	30 (85.7)		
No	58	31 (53.4)		

^*^ *p* < 0.05 and ** *p* < 0.01

### Cell culture and transfection

The human CC cell lines (SiHa, Hela and C33A) and human normal cervical cell line HcerEpic were purchased from the ATCC and cultured in our laboratory. HcerEpic was grown in RPMI-1640 culture medium. And CC cells HeLa, SiHa and C33A were cultured in DMEM medium. The medium was supplemented with 10% fetal bovine serum (FBS), 1% glutamine and 1% penicillin–streptomycin。The cells were cultured in a incubator containing 5% carbon dioxide at 37 ℃.

Three different G6PC siRNAs are established by RiboBio (Guangzhou, China), including si-G6PC-1, si-G6PC-2 and si-G6PC-3. The sequence of si-G6PC-1, si-G6PC-2 and si-G6PC-3 were 5′-GCTGAATGTCTGTCTGTCA-3′, 5′-CGTCCATACTGGTGGGTTT-3′ and 5′-CCATCTGGTTCCATCTTCA-3′. The cells were incubated with Opti-MEM (Reduced Serum Medium, Gibco) containing premixed siRNA (30 nM) and 5 uL of Lipofectamine 2000 (Invitrogen, Carlsbad, CA) according to the manufacturer’s instructions. Lentiviral of overexpression G6PC were constructed by Cyagen Biosciences. According to the manufacturer’s requirements, the cells were infected with the lentiviral vector for 6 h, and the medium was replaced with fresh medium and kept for 72 h. Then, the G6PC-transduced cells were incubated with 1 μg/Ml puromycin in the medium for 1 week to select stably transfected cells.

### Western blot

The treated cells were collected and lysed. After quantification and denaturation, 8%—10% SDS-PAGE was used to isolate the protein and transferred to a PVDF membrane. 5% skimmed milk was used to block the membrane for 2 h, and probed with the primary antibody overnight at 4 °C. The next day, the secondary antibody was detected at room temperature for 2 h. Enzyme linked chemiluminescence (ECL) detection was carried out according to the manufacturer's agreement. Use chemiluminescence and fluorescence imaging systems to quantitatively analyze the results.

### Immunohistochemistry (IHC)

IHC analysis was performed using DAKO LSAB kit (DAKO A/S, Glostrup, Denmark). Tissue specimens were fixed and stained using G6PC antibody as described previously [11]. The score of tissue specimens was established by pathologists who did not possess knowledge of the clinical data. Briefly, the dyeing intensity was 0 (no staining), 1 (light yellow), 2 (brown yellow) and 3 (tan). Percentage of positive cells was 0 (< 10%), 1 (10–25%), 2 (26–50%), 3 (51–75%), 4 (76–100%). The multiplication of the staining intensity and the percentage of stained positive cells is the positive grade, 0 (-), 1–4 ( +), 5–8 (+ +), 9–12 (+ + +). And (-) is regarded as negative, ( +) is regarded as weak positive, (+ +) and (+ + +) are regarded as strong positive expression. For survival analysis, ‘ − ’, ‘ + ’scored samples were considered as low G6PC expression, and ‘ +  + ’, ‘ +  +  + ’ scored samples were considered as high G6PC expression.

### MTT and Colony Formation Assays

The cells were seeded in a 96-well plate at a concentration of about 5000 cells per well and cultured. After 24 h, 48 h, 72 h, 96 h and 120 h, remove the medium and add MTT solution, and incubate in 37℃ incubator for 4 h. Then, DMSO was added and detected on a full-wavelength spectrophotometer (Tecan, Switzerland).

For colony formation, 1000 cells were seeded in 6-well plates. After 2 weeks, when colonies were visible, they were fixed with methanol and stained with Giemsa.

### Wound healing assay

The cells are seeded on 6-well plate, and when the cells are 80% confluent, a cell wound is created by scraping the cells with the tip of a micropipette. The medium was then changed immediately, and spontaneous cell migration was monitored at 0 and 48 h using a microscopy (Olympus, Japan).

Cell migration and invasion assays.

The migration and invasion abilities of cells were assessed using non-Matrigel-coated and Matrigel-coated Transwell inserts (BD Biosciences, San Diego, CA, USA), respectively. For cell migration, 5 × 10^4^ cells were added to the upper chamber, fibronectin-free medium was added to the lower chamber, and the cells were incubated at 37 °C for several hours. For cell invasion, 1 × 10^5^ cells were added to the upper chamber, medium containing fibronectin (20 μg/mL) was added to the lower chamber, and the cells were incubated at 37 °C for several hours. Migrated or invaded cells (on the lower side of the membranes or in the lower well) were then fixed in 4% paraformaldehyde for 20 min and stained with hematoxylin for 15 min. The cells were counted under a microscope.

### Tube formation assay

Matrigel (BD Biosciences, San Diego, CA, USA) was diluted 1:1 in serum-free cell culture medium. Before cell seeding, add to 96-well plate and cure at 37 °C for 4 h. HUVEC was resuspended in FBS-free medium containing cancer cell culture supernatant and seeded on polymerized Matrigel. After incubating for 3 h at 37 °C, the tubular networks were visualized with microscope. The conditioned medium was: HeLa and SiHa cells were treated under different transfection conditions and the medium collected after 48 h.

### Animal studies

In order to evaluate the effect of G6PC on the tumorigenicity in vivo, Siha cells stably overexpressing G6PC and transfected with si-control or si-G6PC#1 was implanted subcutaneously in the left or right flank of 5-week-old BALB/c nude female mice (Vital Rivers, Beijing, China) to establish tumour model. After 5 weeks, all mice were sacrifices, then tumors were removed, and the average tumor weight was measured. All experiments were performed in keeping with the procedures and protocols of the Animal Ethics Committee of Yanbian University.

### Chick chorioallantoic membrane assay (CAM assay)

The fertilized eggs are kept in an incubator with a temperature of 37 °C and a humidity of 60–70%. After 8 days, the cells were added to the sterile rubber ring on the exposed CAM, an artificial balloon was established under aseptic conditions and sealed with tape. After another 48 h incubation, CAMs were photographed with a microscope (Olympus BX51).

### Immunofluorescence (IF)

The secondary antibodies we used were Alexa fluor 488 and Alexa fluor 568, respectively. The details of the experiment are described previously [12].

### Statistical analysis

The data used SPSS 26.0, graphpad prism 8.0 and JMP software for statistical analysis. Chi-square test and Fisher’s exact test were used to evaluate the correlation between G6PC expression and clinicopathological features. Kaplan–Meier method was used to analyze survival curve. We also did the univariate logistic regression analysis and multivariable logistic analysis. Group comparisons for continuous data were done by t-test for independent means or one-way ANOVA. Each experiment was repeated at least three times. Data are shown as mean ± SD. In all these tests, *P* < 0.05 was considered statistically significant.

## Results

### G6PC expression was upregulated in CC and correlated with a poor prognosis

**Fig. 1:**
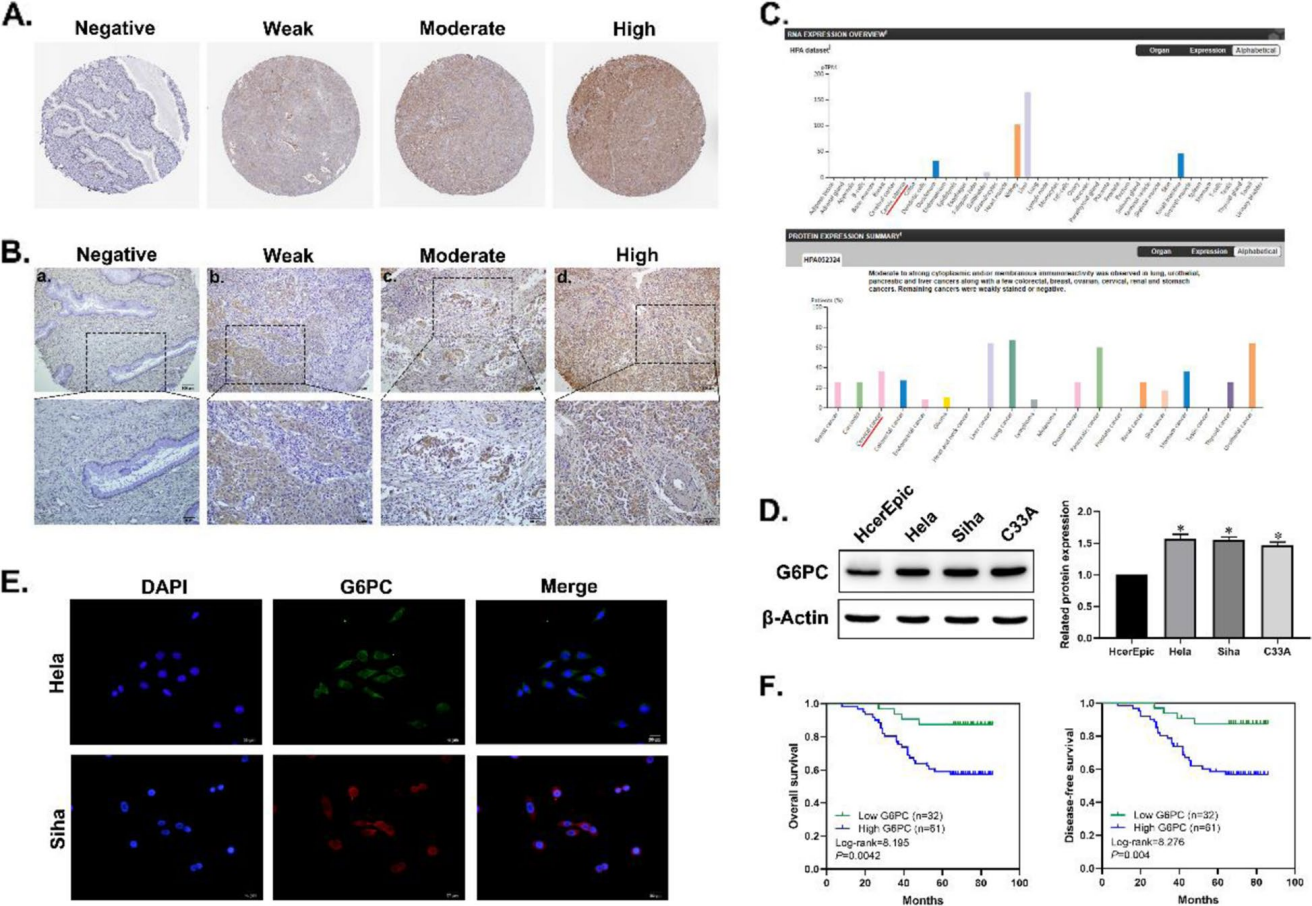
G6PC expression was upregulated in CC and correlated with a poor prognosis. (**A**) Immunohistochemistry of G6PC proteins in clinical specimens from the human protein atlas (www.proteinatlas.org). (**B**) The expression of G6PC in Cervical tissue samples was detected by immunohistochemistry. a: Negative G6PC expression in adjacent normal cervical tissue. b: Weak G6PC expression in CC tissue. c: Moderate G6PC expression in CC tissue. d: Strong G6PC expression in CC tissue. (**C**) Overview of G6PC expression levels in CC tissues and normal cervical tissues in HPA database. (**D**) Western blot assays performed on CC cell lines as indicated. (**E**) Immunofluorescence staining of the G6PC protein in Hela and Siha cells; a cytoplasmic staining pattern was also observed. (**F**) Overall survival rates and Disease-free survival rates of cervical cancer patients in different expression level of G6PC analyzed by Kaplan–Meier

To analyze the expression of G6PC in human CC, we first obtained information about changes in G6PC protein expression in clinical specimens from the Human Protein Atlas (Fig. 1A). Next, we performed IHC staining to analyze the expression of G6PC in 93 CC tissues and 24 adjacent normal cervical tissues. As shown in Fig. 1B, G6PC was predominantly located in the cytoplasm of cancer cells and remarkably elevated in CC tissues, and the positive staining rates and strongly positive staining rates of G6PC in cervical cancer tissues are 83.9% (78/93) and 65.6% (61/93), respectively (Table 1). And in the HPA database, G6PC expression was hardly detected in normal sections, but there were significantly higher levels in CC (Fig. 1C), indicating that G6PC protein potentially plays an important role in CC development. Moreover, we evaluated the expression profile of G6PC in CC cells. Western blot analysis showed that compared with normal cervical epithelial cells (HcerEpic), HeLa, SiHa and C33A CC cells exhibited significantly upregulated G6PC protein expression (Fig. 1D). Immunofluorescence staining further confirmed that G6PC was mainly expressed in the cytoplasm (Fig. 1E). According to the results of IHC, correlation analysis showed that protein expression level of G6PC was significantly correlated with patient LN metastasis(*P* = 0.021), clinical stage(*P* = 0.007) and recurrence(*P* = 0.002). However, the expression level of G6PC was not related to age, tumor invasion depth or histological grade (Table 2).

Next, we further investigated the prognostic value of G6PC expression in CC. We analysed the data of 93 patients using Kaplan–Meier. The results revealed that the overall survival and disease-free survival were all correlated with the expression of G6PC (*P* = 0.0042, *P* = 0.004, respectively) (Fig. 1F), and the patients with high level of G6PC expression showed poorer overall survival and disease-free survival than those with low level of G6PC expression. Further analysis of the Cox proportional hazards model revealed that the age(*P* = 0.000), LN metastasis(*P* = 0.000), clinical stage (*P* = 0.000) and G6PC (*P* = 0.009) expression were correlated with OS rates. Moreover, multivariate Cox analysis confirmed that G6PC could be a significant independent prognostic factor in CC (Table 3).

**Table 3:** Univariate and multivariate survival analysis of clinicopathological features in 93 cervical cancer

**Characteristics**	**B**	**SE**	**Wald**	**HR**	**95%CI**		***P* value**
					**Lower**	**Upper**	
**Univariate survival analyses**							
Age	0.158	0.024	42.039	1.171	1.117	1.228	0.000**
Histological grade	0.163	0.413	0.156	1.177	0.524	2.645	0.693
Clinical stage	1.043	0.228	20.915	2.838	1.815	4.438	0.000**
LN metastasis	1.360	0.375	13.181	3.897	1.870	8.121	0.000**
G6PC	1.413	0.537	6.915	4.110	1.433	11.784	0.009**
**Multivariate survival analyses**							
Age	0.148	0.025	34.940	1.159	1.104	1.218	0.000**
Clinical stage	1.018	0.465	4.786	2.768	1.112	6.892	0.029*
G6PC	1.151	0.577	3.980	3.161	1.020	9.790	0.046*

Statistical analyses were performed using Cox proportional hazard regression model. *P < 0.05, ** *P* < 0.01

### G6PC promotes cell proliferation and tumorigenesis of CC

**Fig. 2:**
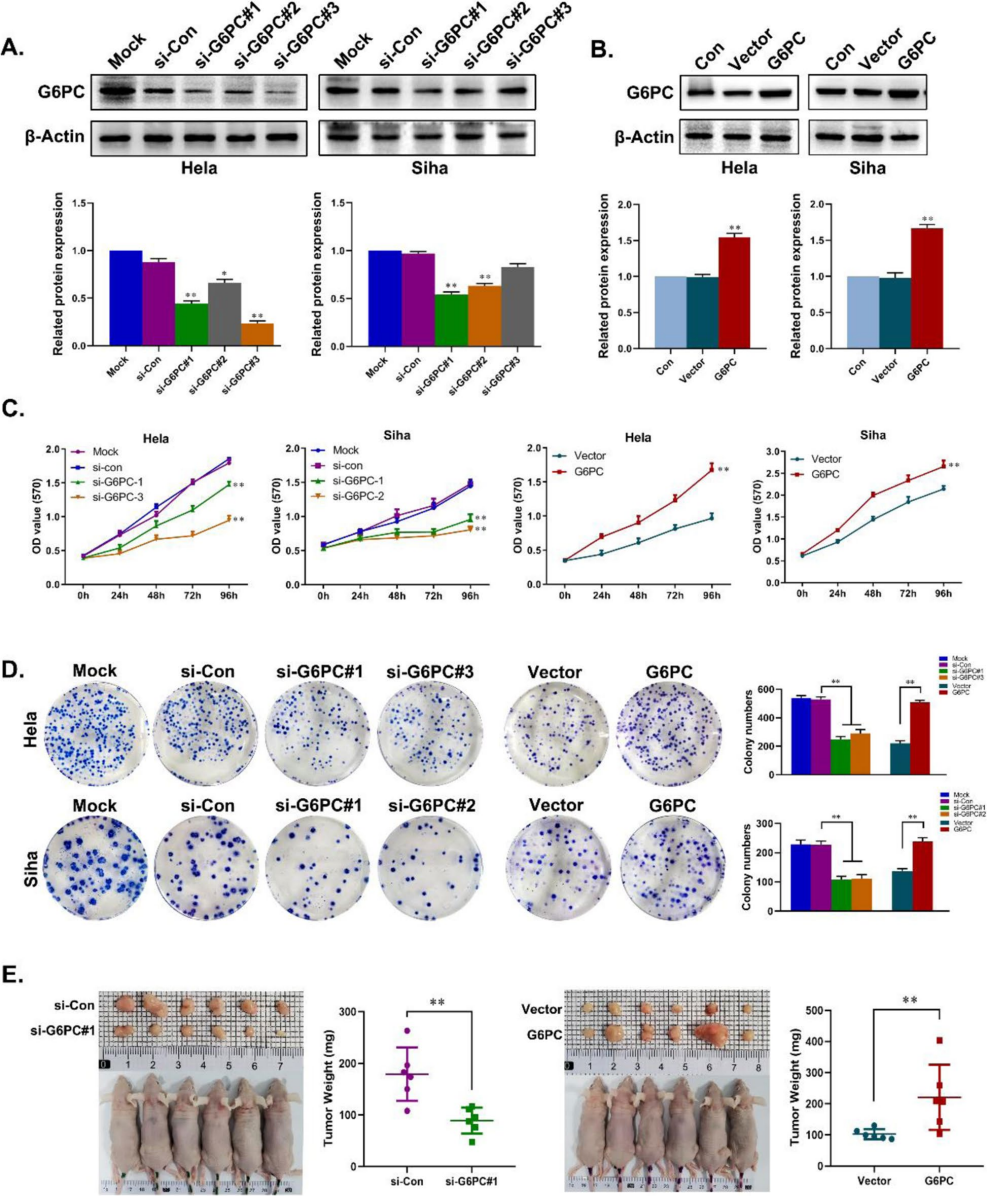
G6PC promotes CC cells proliferation in vitro and in vivo. (**A**, **B**) G6PC expression level in the constructed Hela and Siha cells was examined by Western blot. β-Actin was used as a loading control. ^*^P < 0.05, ^**^P < 0.01. (**C**, **D**) Cell proliferation and colonization was confirmed by MTT (**C**), colony (**D**). ^*^P < 0.05, ^**^P < 0.01. (**E**) Indicated that cells were injected into the nude mice. The tumors were dissected at day 35 and weighted

We further explored the effect of G6PC on the biological behavior of CC. The depletion or overexpression of G6PC in HeLa and SiHa cells was confirmed by Western blot (Fig. 2A,B). MTT and colony formation analysies showed that G6PC depletion significantly inhibited cell proliferation and clonogenicity, while G6PC overexpression enhanced cell proliferation and clonogenicity (Fig. 2C,D). In vivo, the effect of G6PC on tumorigenesis was studied with a xenograft mouse model. Compared with the si-Con group, the weights of tumors that generated from the G6PC-knockdown cells were markedly decreased. When G6PC was highly expressed, the tumor weights significantly increased (Fig. 2E). These results indicate that G6PC can promote cell proliferation and tumorigenesis, suggesting that G6PC may play an important role in the progression of CC.

### Effect of G6PC on migration, invasion and epithelial-mesenchymal transition (EMT) in CC

**Fig. 3:**
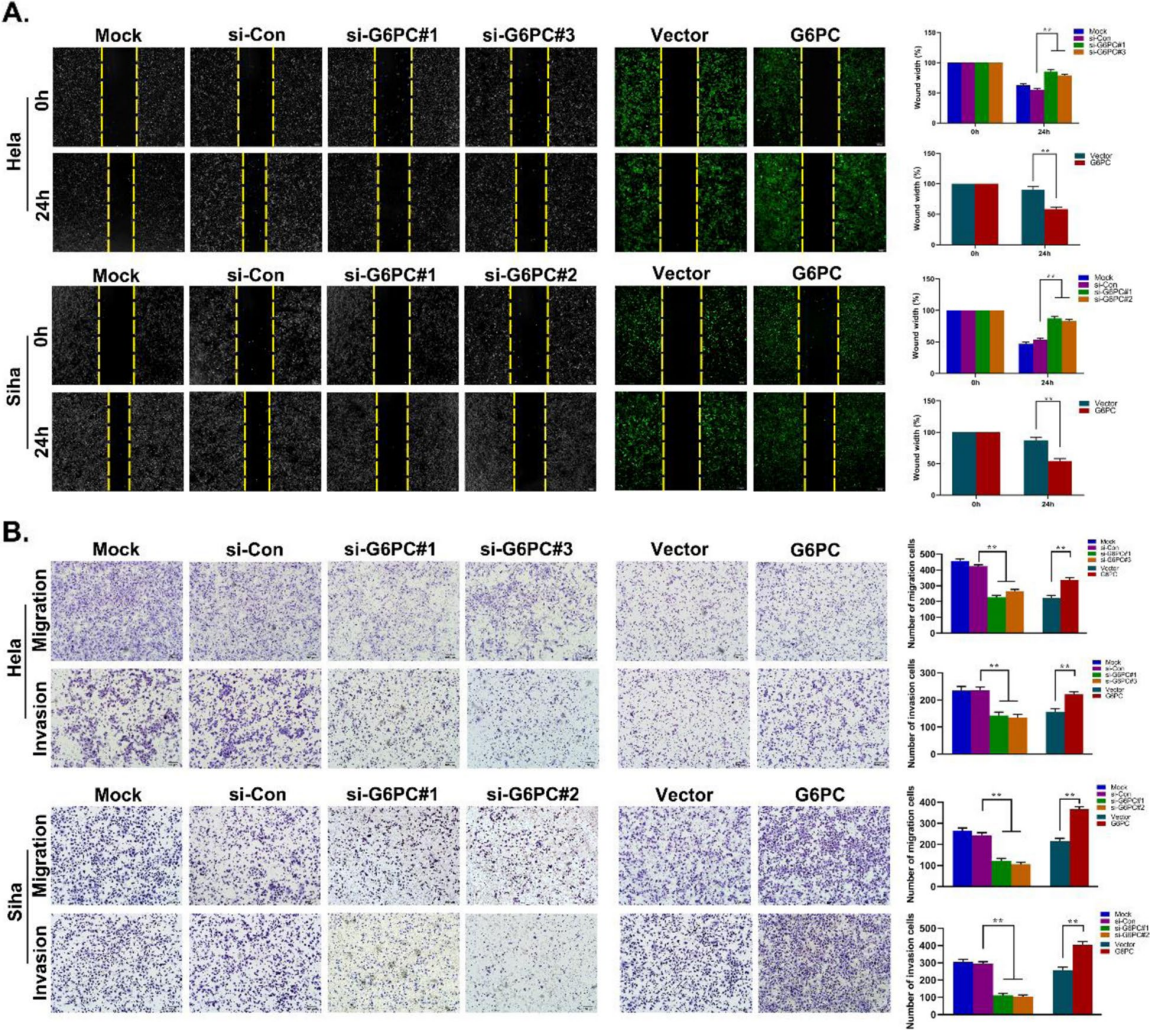
G6PC promotes CC metastasis and invasion. (**A**, **B**) Migration and invasion capacity of G6PC in Hela and Siha cells was examined by wound healing assay (**A**), transwell migration and invasion assay (**B**). ^*^P < 0.05, ^**^P < 0.01

**Fig. 4:**
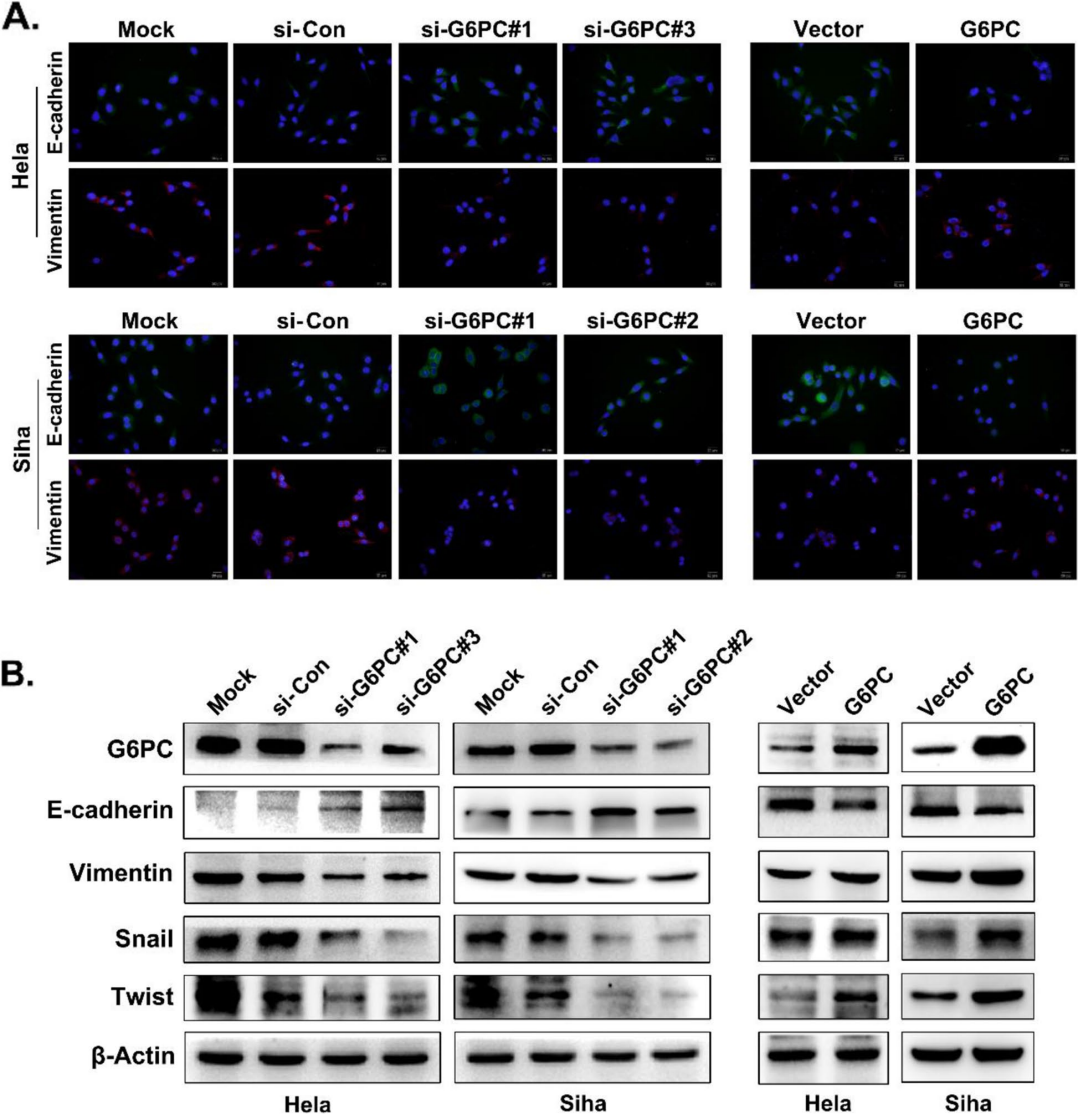
G6PC promotes EMT in CC cells. (**B**) EMT markers were confirmed by Western blot in the constructed Hela and Siha cells. β-Actin was used as the loading control. (**A**) Immunofluorescence staining for EMT markers in the constructed Hela and Siha cells

Then, we studied the role of G6PC in the migration and invasion abilities of CC cells with wound healing and Transwell assays. When G6PC was overexpressed, the wound healing ability of CC cells was significantly increased, and when G6PC expression was silenced, the wound healing ability of CC cells was inhibited (Fig. 3A). Transwell analysis further proved that knockdown of G6PC significantly disrupted the cell migration and invasion capabilities, while overexpression of G6PC significantly enhanced these capabilities (Fig. 3B). In light of the close relationship between EMT and tumor migration, and considering the impact of G6PC on cell invasion and metastasis, we hypothesized that G6PC could affect the EMT of CC. The Western blot results showed that depletion of G6PC significantly increased the expression of E-cadherin, while the expression levels of Vimentin, Snail and Twist were decreased. Conversely, G6PC overexpression reduced E-cadherin expression and upregulated the expression of Vimentin, Snail and Twist (Fig. 4A). In addition, immunofluorescence further verified the effect of G6PC in promoting EMT (Fig. 4B).

### G6PC promotes CC cells angiogenesis in vitro and in vivo

**Fig. 5:**
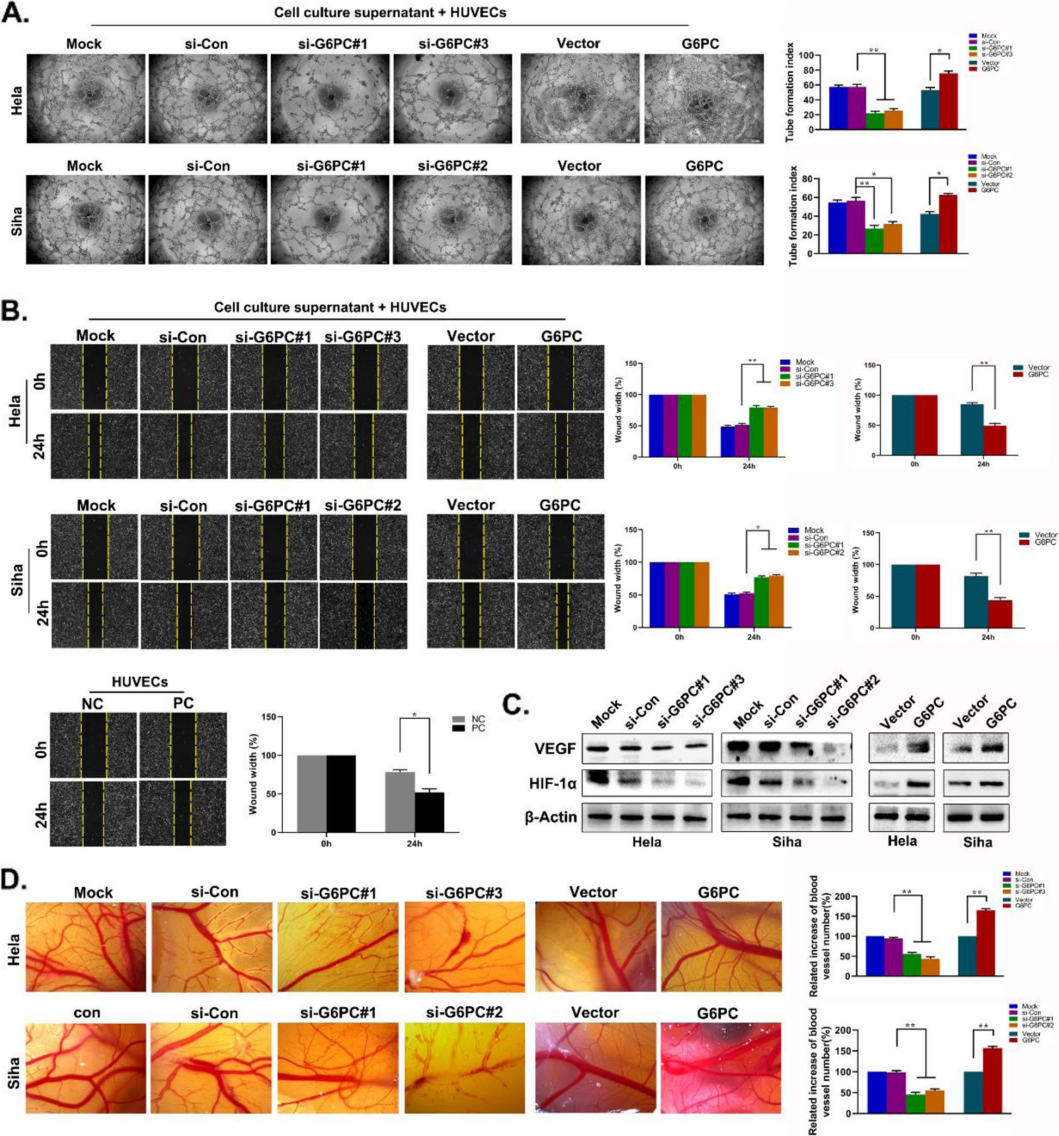
G6PC promotes tumour angiogenesis in vitro and in vivo. (**A**) Tube formation assay was performed in Hela and Siha cells. ^*^P < 0.05, ^**^P < 0.01. (**B**) The HUVEC migration experiment was used to detect the effect of differential expression of G6PC on the migration ability of vascular endothelial cells. NC(negative control): serum-free culture medium; PC(positive control): whole serum culture medium. ^*^P < 0.05, ^**^P < 0.01. (**C**) Western blot analysis of VEGF and HIF-1α in the constructed Hela and Siha cells. β-Actin was used as the loading control. (**D**) CAM assays were performed to confirm the effect of G6PC on tumour angiogenesis ex vivo. ^*^P < 0.05, ^**^P < 0.01

Since angiogenesis is critical for cancer metastasis and progression, we hypothesized that G6PC plays a role in CC angiogenesis. Through the microtubule formation assay, we observed that the microtubule formation ability of HUVEC was decreased in G6PC depleted cells, but increased in G6PC overexpressing cells (Fig. 5A). HUVEC wound healing assay results showed that G6PC significantly promoted the migration ability of HUVECs (Fig. 5B). Further Western blot analysis showed that the silencing of G6PC expression reduced the expression levels of VEGF and HIF-1α, and the overexpression of G6PC increased the expression levels of these proteins, indicating that G6PC may have proangiogenic properties in CC (Fig. 5C). We also assessed the effect of G6PC on angiogenesis in vivo with a CAM assay, and found that the downregulation of G6PC expression reduced the angiogenesis of CC cells. In contrast, the upregulation of G6PC expression increased the angiogenesis of CC cells (Fig. 5D). These results demonstrate that G6PC can regulate angiogenesis in CC.

### G6PC exerts its CC tumor-promoting effect by activating the AKT/mTOR signaling pathway

**Fig. 6:**
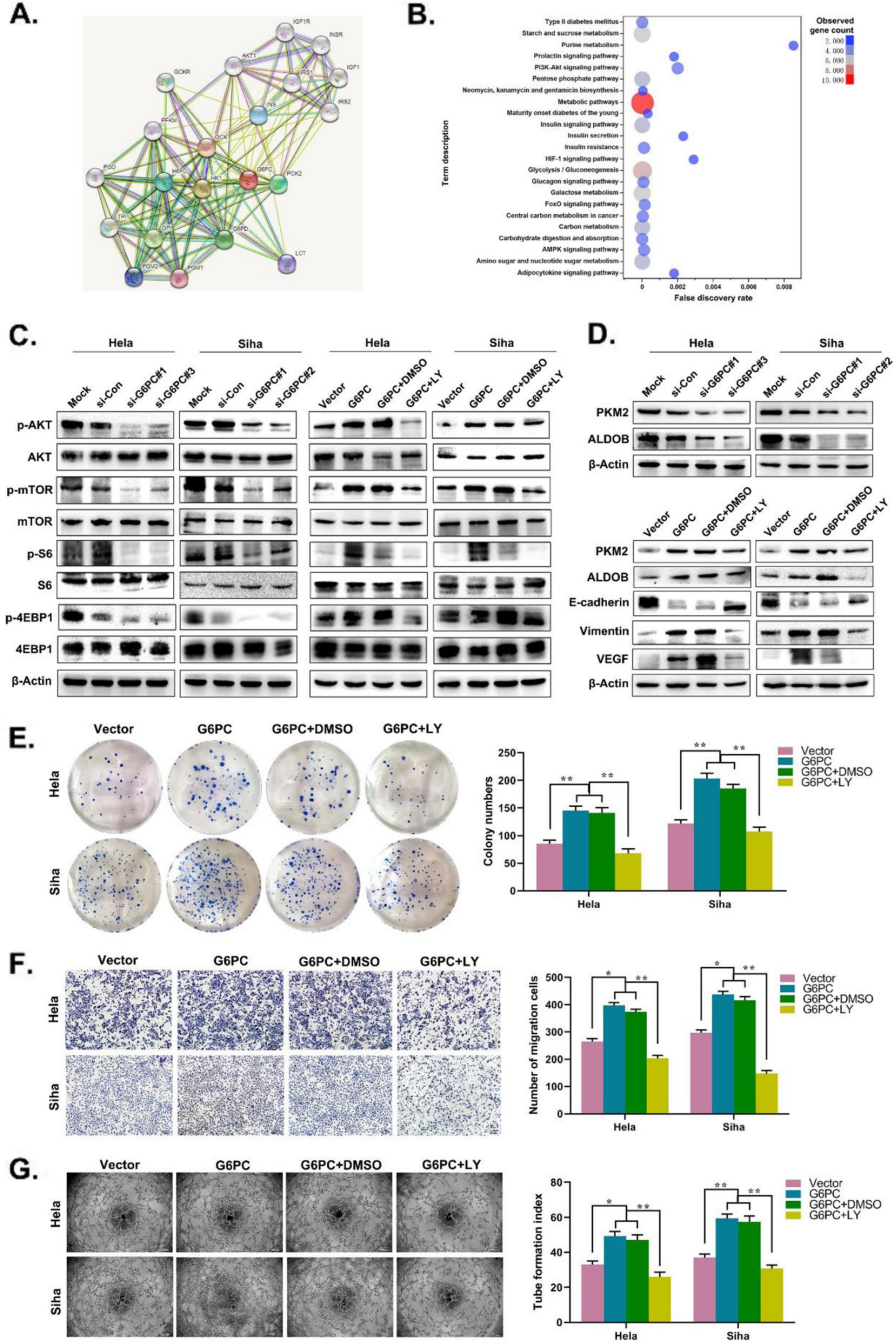
G6PC overexpression promotes CC progression via AKT/mTOR pathway. (**A**) Protein association network of G6PC that were generated with Search Tool for the Retrieval of Interacting Genes (STRING: https://string-db.org/). (**B**) G6PC-related KEGG pathways in CC tissue in String database. (**C**) Western blot analysis of t-AKT/p-AKT((Ser473), t-mTOR/p-mTOR, t-S6/p-S6 and t-4EBP1/p-4EBP1 in the constructed cells. β-Actin was used as the loading control. The protein expression levels of indicated genes were also analysed in G6PC overexpressed cells treated with PI3K inhibitor LY294002 (20 μmol/l). (**D**) Western blot analysis of PKM2 and ALDOB in the constructed cells. β-Actin was used as the loading control. The protein expression levels of indicated genes were also analysed in G6PC overexpressed cells treated with PI3K inhibitor LY294002 (20 μmol/l). (**E**–**G**) Representative images showing the colony formation (**D**) Migration assay (**E**) and Tube formation assay (**F**) of Hela and Siha cells overexpressing G6PC or treated with LY294002

The PI3K/AKT/mTOR pathway is one of the major signaling pathways that has been shown to be important in cancer and is involved in a variety of cell functions, including proliferation, growth, glucose metabolism, differentiation and EMT [13]. STRING database analysis showed that G6PC has an interactive relationship with AKT and glycolysis-related enzymes (Fig. 6A). Further analysis of publicly available KEGG pathway data showed that G6PC is related to the PI3K/AKT/mTOR signaling pathway and glycolysis (Fig. 6B). Therefore, we investigated whether abnormal expression of G6PC can affect the PI3K/AKT/mTOR pathway and glycolysis in CC cells by Western blot. The results showed that the levels of p-AKT, p-mTOR, p-S6, and p-4EBP1 were decreased in G6PC-knockdown CC cells, but increased in G6PC-overexpressing cells; meanwhile, the levels of t-AKT, t-mTOR, t-S6 and t-4EBP1 were not significantly changed. Consistent with these findings, we also found that the PI3K inhibitor LY294002 significantly reversed the G6PC overexpression-induced increase in the levels of p-AKT, p-mTOR, p-S6 and p-4EBP1. (Fig. 6C). On the other hand, the expression levels of key enzymes of glycolysis PKM2 and ALDOB were downregulated by G6PC silencing, and upregulated by G6PC overexpression. In addition, LY294002 also caused the downregulation of Vimentin, PKM2, ALDOB and VEGF, and the up-regulation of E-cadherin in CC cells overexpressing G6PC (Fig. 6D). A series of functional experiments revealed that LY294002 attenuated the proliferation, migration, and angiogenesis abilities of G6PC-overexpressing cells (Fig. 6E-G). In summary, these results indicate that G6PC promotes the progression of CC at least in part via the AKT/mTOR pathway.

## Discussion

G6PC is a key enzyme of gluconeogenesis and is mainly expressed in the liver and kidney. G6PC deficiency can cause a serious metabolic disease called glycogen storage disease type I (GSD-1), which is characterized by impaired blood glucose homeostasis and metabolic disorders and can further progress to cancer, including hepatocellular carcinoma and renal neoplasia [14, 15]. A previous study showed that G6PC is underexpressed in hepatocellular carcinoma and inhibits tumor growth, suggesting that G6PC may act as a tumor suppressor [7]. However, it has been reported that G6PC expression is upregulated in glioblastoma and ovarian cancer, and this upregulated G6PC expression promotes cell proliferation and an aggressive phenotype, indicating that G6PC may also play a role as an oncogene [9, 10]. Therefore, different effects of G6PC have been observed in different cancers, and it has important biological functions other than its role in gluconeogenesis these other functions are worthy of further study.

To determine the potential function of G6PC in the pathogenesis of CC, we first assessed the expression of G6PC in CC tissues through IHC, and we found that the level of G6PC in CC tissues was significantly higher than that in normal cervical tissues. The same results were obtained in CC cells, which was consistent with the findings by Guo et al. in ovarian cancer [9]. We also found that G6PC was predominantly located in the cytoplasm of cancer cells. More importantly, our clinical data analyses showed that G6PC overexpression was significantly correlated with aggressive cancer features in CC, such as LN metastasis, clinical stage and recurrence. Of note, G6PC overexpression was significantly related to poor prognosis of CC patients. Therefore, we speculate that G6PC may play a role in the progression of CC and become a novel predictor of the cervical cancer.

Cancer cell proliferation underlies tumorigenesis and metastatic dissemination, which are the main problems in cancers. Next, we considered whether G6PC is involved in the proliferation of cervical cancer cells. We found that G6PC overexpression promotes the proliferation of tumor cells, which was also verified by in vivo experiments. EMT is a well-characterized embryological process that helps enhance the mobility and invasiveness of cancer cells, which is an important step in tumor metastasis [16, 17]. Previous studies have shown that G6PC can promote the metastasis of glioblastoma [10], but the mechanisms by which EMT are regulated are still unknown. In this study, we found that G6PC significantly promoted the migration and invasion of CC cells in vitro. Furthermore, we found that G6PC knockdown significantly increased the expression of the epithelial marker E-cadherin and decreased the expression of the mesenchymal marker Vimentin, while G6PC overexpression led to the opposite results. In addition, the transcription factors Snail and Twist have been shown to be involved in the G6PC-mediated regulation of EMT. These data indicate that G6PC inhibits the migration of CC cells by regulating the progression of EMT.

Angiogenesis is necessary for continuous tumor growth and promotes the distant metastasis of tumor cells [18, 19]. According to reports, dexamethasone regulates HCC growth and angiogenesis by enhancing the expression of G6PC and PEPCK [20]. In addition, G6PT, another component of the G6Pase complex, has also been reported to be closely related to the regulation of microvascular formation and migration [21, 22]. In this study, we found that silencing G6PC inhibited the formation of microtubules and the migration of HUVECs in vitro and reduced the number of vessel branches formed ex vivo. In addition, the Western blot analysis results also showed that G6PC knockdown reduced the expression levels of HIF-1α and VEGF, while G6PC overexpression increased the expression levels of these two proteins. In summary, our data, based on previous reports, indicate that G6PC plays a vital role in CC angiogenesis.

The PI3K/AKT/mTOR signaling pathway plays an important role in the malignant progression of tumors, regulating many cell processes including proliferation and metastasis [23–25]. Sara Abbadi et al. found that G6PC can activate the AKT pathway [10]. In this study, the knockdown of G6PC in Hela and Siha cells reduced the expression of p-AKT, p-mTOR, p-4EBP1 and p-S6, and the opposite results were observed when G6PC was overexpressed. These findings show that G6PC affects CC at least in part through the AKT pathway, and the addition of PI3K inhibitor LY294002 further confirmed our conclusion. Due to its antagonism of glycolysis, the gluconeogenesis pathway is usually inhibited in cancer. However, the expression of enzymes related to gluconeogenesis is not necessarily consistent with the level of gluconeogenesis, because certain types of cancer may use abbreviated gluconeogenesis to support their biosynthetic requirements, thereby promoting metabolic flexibility [26, 27]. Our results showed that the silencing of G6PC could attenuate the expression of glycolysis-related enzymes, while overexpression of G6PC increased their expression, suggesting that G6PC played positive roles in CC mostly or partially throughout glycolysis promotion. The PI3K/AKT signaling and its mammalian mTOR target are key regulators of glycolysis reprogramming. Studies have shown that PI3K/AKT signaling can increase the expression of glucose transporter (GLUT1) and enhance glucose uptake. In this study,the enhanced glycolysis induced by G6PC overexpression were abrogated by inhibition of PI3K/AKT pathway. Our results indicated that G6PC likely promoted aerobic glycolysis, cell proliferation and metastasis by regulating the activation of AKT/mTOR pathway, and the specific mechanism requires further research.

At present, most of the research reports on G6PC are related to metabolism, which is not comprehensive in the field of cancer. This study is the first to analyze the expression of G6PC and its role in the occurrence and development of CC from multiple levels of bioinformatics analysis, tissues, cells and animals, and initially explore its mechanism of action. Herein we have shown that overexpressed G6PC promotes the malignant phenotype of CC cells and the PI3K/AKT/mTOR pathway plays an important role in the process of G6PC acting as a proto-oncogene. These results provide a new theoretical basis for G6PC as an early diagnostic marker and therapeutic target for CC. Last but not least, our research also has certain limitations. Enlarged sample size of CC tissues and transgenic animal models are being established to verify the regulatory mechanism of G6PC in CC. Further studies on the role of G6PC in CC will lead to more selective and efficient anticancer effects.

## Conclusions

**Fig. 7:**
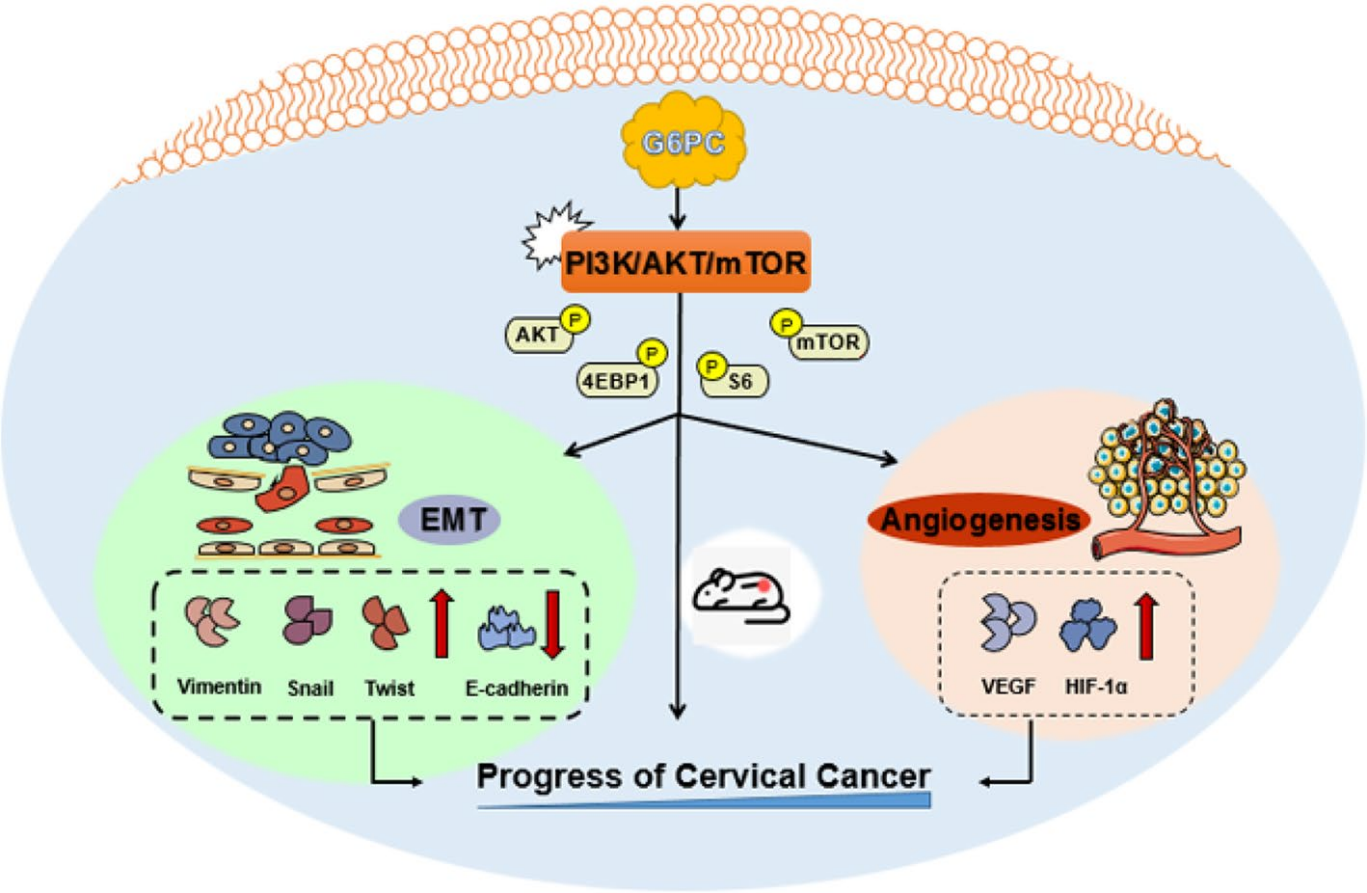
Simplified schematic diagram indicates the potential role of G6PC in regulating the progression by AKT/mTOR pathway in CC

In summary, our observation suggested that G6PC plays a key role in the progression of CC, and overexpressed G6PC is closely related to patient LN metastasis, clinical stage, recurrence and shortened survival. In addition, we supplied a conceptual framework to illustrate how G6PC promoted the proliferation and metastasis of CC cells and linked the molecular mechanisms (Fig. 7). Therefore, this study links G6PC to CC progression and establishes G6PC as a potential biomarker for a potential therapeutic target.

## Data Availability

All data generated or analyzed during this study are included in this published article.

## Abbreviations

G6PC: Glucose-6-phosphatase catalytic subunit
EMT: Epithelial mesenchymal transition
CC: Cervical cancer
HPV: Human papillomavirus
AJCC: American Joint Committee on Cancer
FBS: Fetal bovine serum
ECL: Enzyme linked chemiluminescence
DMEM: Dulbecco’s modified Eagle’s medium
siRNA: Small interfering RNA
NC: Negative control
IHC: Immunohistochemistry
CAM: Chick chorioallantoic membrane assay
IF: Immunofluorescence
GSD-1: Glycogen storage disease type I
SPSS: Statistical Package for Social Sciences
AKT: Protein kinase B
KEGG: Kyoto Encyclopedia of Genes and Genomes
